# Altered mitochondrial quality control in Atg7-deficient VSMCs promotes enhanced apoptosis and is linked to unstable atherosclerotic plaque phenotype

**DOI:** 10.1038/s41419-019-1400-0

**Published:** 2019-02-11

**Authors:** Hripsimé Nahapetyan, Manon Moulis, Elisa Grousset, Julien Faccini, Marie-Hélène Grazide, Elodie Mucher, Meyer Elbaz, Wim Martinet, Cécile Vindis

**Affiliations:** 1 Inserm, UMR 1048, Institut des Maladies Métaboliques et Cardiovasculaires, F-31342 Toulouse, France; 2 Université de Toulouse III, F-31342 Toulouse, France; 3 Fédération des Services de Cardiologie, Hôpital Rangueil, F-31342 Toulouse, France; 4 Laboratory of Physiopharmacology, University of Antwerp, Antwerp, Belgium

## Abstract

Vascular smooth muscle cells (VSMCs) are one of the main cellular determinants in arterial pathology. A large body of evidence indicates that death of VSMCs is associated with features of high-risk/vulnerable atherosclerotic plaques. Mitochondrial turnover is an essential aspect of the mitochondrial quality control in which dysfunctional mitochondria are selectively eliminated through autophagy and replaced through expansion of preexisting mitochondria. Even though successful autophagy promotes VSMC survival, it is unclear whether reduced autophagic flux affects mitochondrial quality control of VSMCs in atherosclerotic plaques. By using apolipoprotein E-deficient (ApoE^−/−^) mice carrying a VSMC-specific deletion of the essential autophagy gene *Atg7*, we show in the present study that impaired VSMC autophagy promotes an unstable plaque phenotype, as well as the accumulation of fragmented mitochondria with reduced bioenergetic efficiency and more oxidative stress. Furthermore, we demonstrate that disrupted autophagic flux is linked to defective mitophagy and biogenesis of mitochondria, which exacerbate VSMC apoptosis and in turn plaque vulnerability. Overall, our data indicate that mitochondrial quality control is a promising therapeutic target to stabilize atherosclerotic plaques.

## Introduction

Vascular smooth muscle cells (VSMCs) regulate various aspects of vessel homeostasis, including contraction, dilation, and vessel remodeling. Consequently, VSMCs are one of the main cellular determinants of arterial wall pathology. In cardiovascular disease (CVD), such as atherosclerosis, VSMCs are the first to support lipid retention and lipid overload through foam cell formation and death^1^. In stable atherosclerotic lesions, VSMCs are predominant in the fibrous cap and contribute to its thickness by producing matrix components. Conversely, induction of VSMC apoptosis gives rise to thinner fibrous caps and reduced matrix protein deposition. In addition, VSMC apoptosis contributes to enhanced atherosclerotic plaque vulnerability and medial degeneration, and promotes plaque thrombogenicity by exposing phosphatidylserine on the surface of apoptotic cells^2^. Thus, VSMC apoptosis recapitulates most of the features of plaque vulnerability such as increased necrotic core formation and plaque inflammation^3^.

However, the mechanisms involved in plaque stability and plaque rupture are complex and the oxidizing and inflammatory environment generated by the presence of pro-atherogenic factors (low-density lipoprotein (LDL) and oxidized lipids, oxidative stress, cytokines) can trigger pro-survival and pro-death processes, which are concomitantly activated in cells. The outcome (life vs death) depends on the balance between these pathways. Besides apoptosis, a growing body of evidence indicates that induction of autophagy plays an important role in the response of VSMCs to various atherogenic stressors, such as oxidized lipids^4^. Autophagy is a crucial cellular catabolic process that is responsible for the destruction of long-lived proteins and organelles via a lysosome-dependent pathway. In the general form of the process, cytoplasmic cargo targeted for destruction is sequestered inside double-membrane vesicles called autophagosomes and is delivered to the lysosome by fusion for breakdown. The degradation products are then transported back to the cytoplasm where they can be reused for biosynthesis or energy production. This process is important for maintaining cellular homeostasis and deregulated autophagy has been implicated in the pathogenesis of a wide array of diseases^5^. Variations in the level of autophagy can have major effects on vessel wall function and the initiation or progression of vascular diseases, including atherosclerosis^6^. Several autophagy stimuli are present within the developing plaque, such as inflammatory cytokines, reactive oxygen species (ROS), oxidized lipid species, growth factors, and metabolic stressors^7^. Recently, autophagy deficiency in murine VSMCs was shown to lead to accelerated senescence and it also promoted ligation-induced neointima formation, thus implying autophagy in the control of VSMC phenotype and proliferation^8^. Although most altered organelles and cellular structures can be eliminated by autophagy as a general clearance system^9^, damaged mitochondria can also be discarded by a specific form of autophagy known as mitophagy or mitochondrial autophagy^10^. Mitophagy can occur in specific developmental processes, such as the maturation of erythrocytes^11^, and also following pathological mitochondrial damage to eliminate altered mitochondria and to prevent cell death^12^. Indeed, during the early stages of apoptosis, alterations in mitochondrial morphology (fragmentation and remodeling) and function (decline in mitochondrial membrane potential and increase in radical production) are observed. All these events are known to be prerequisites for the initiation of mitophagy^13,14^. Selective elimination of dysfunctional mitochondria reduces the release of cytochrome c and other pro-apoptotic factors into the cytosol that activate downstream cell death pathways^15^.

Using loss- or gain-of-expression approaches, we recently demonstrated that mitophagy has critical consequences on VSMC fate by enhancing apoptosis or by favoring cell survival. For example, knockdown of Phosphatase and tensin homolog PTEN-induced putative kinase 1 (PINK1) or E3 ubiquitin ligase Parkin by small-interfering RNAs increases the cytotoxic response of human VSMCs challenged with atherogenic lipid stressors, whereas PINK1 or Parkin overexpression has cytoprotective effects^16^. However, balancing mitophagy and mitochondrial biogenesis is essential for maintaining a healthy population of mitochondria. Therefore, a failure to maintain this critical balance results in a population of dysfunctional mitochondria, leading to pathological consequences. Interestingly, endogenous mitochondrial DNA damage in mouse and human atherosclerotic lesions is associated with significant reductions in mitochondrial copy number and respiration, and cultured human plaque VSMCs show increased mitophagy^17^. In this study, we investigated whether defective autophagy in VSMCs could impact mitochondrial quality control and also whether this affected atherosclerotic plaque phenotype and development.

## Materials and methods

### Reagents and antibodies

Bafilomycin A1 (B1793), carbonyl cyanide m-chlorophenyl hydrazone (CCCP) (C2759), propidium iodide (P4170), oligomycin (O4876), 2-[2-[4(trifluoromethoxy)phenyl]hydrazinylidene]-propanedinitrile (FCCP) (C2920), antimycin A (AA) (A8674), and rotenone (R8875) were purchased from Sigma-Aldrich. SYTO-13 (S7575) was purchased from Thermo Fisher Scientific. The Masson’s Trichrome staining kit was obtained from RAL Diagnostic (RAL-361350-0000). The DeadEnd^TM^ Fluorometric Terminal deoxynucleotidyl transferase dUTP nick end labeling (TUNEL) System was purchased from Promega (G3250). Anti-cleaved-caspase 3 (Asp175) (9664), anti-LC3B (2775), anti-VDAC1 (4661), anti-ATG7 (2631), anti-Beclin 1 (3728), anti-GRP75 (2816), and anti-p-Drp-1 (Ser616) (3455) antibodies were from Cell Signaling Technology. Anti-β-actin (A2228) and anti-SQSTM1/P62 (P0067) were from Sigma-Aldrich. Anti-TOMM 40 antibody (H-300) was from Santa Cruz Biotechnologies. Anti-MOMA 2 antibody (MAB1825) was from Merck Millipore, anti-Parkin (ab15954) and anti-PINK1 (ab74487) were from Abcam. Anti-PGC-1α antibody (NBP1-04676) was from NOVUS Biologicals. Anti-TFEB antibody (A303-673A) was from Bethyl Laboratories. Smooth muscle actin rabbit polyclonal antibody (RB-9010-P) was from Thermo Fisher Scientific. Secondary antibodies conjugated to horseradish peroxidase (HRP) were from Cell Signaling Technology.

### Mouse studies

Experiments were conducted according to the guidelines formulated by the European Community for the experimental use of animals (Directive 201/63/EU) and were approved by the Ethics Committee of INSERM and the French Ministry of Agriculture. *Atg7*^*F/F*^ *Tagln/SM22α Cre*^*+*^ and *Atg7*^*+/+*^ *Tagln/SM22α Cre*^*+*^ mice^8^ were crossbred with apolipoprotein E-deficient (ApoE^−/−^) mice to generate *Atg7*^*F/F*^ *Tagln/SM22α Cre*^*+*^, ApoE^−/−^ and *Atg7*^*+/+*^ *Tagln/SM22α Cre*^*+*^, ApoE^−/−^ mice. The Tagln/SM22α promoter allows VSMC-specific Atg7 deletion through Cre-loxP technology. For atherosclerosis studies, male mice aged 6 weeks were fed a high-fat diet (HFD) (ssniff Spezialdiäten GmbH, TD96335) for 10 (*n* = 11/group) or 18 (*n* = 8/group) weeks. At the end of the experiment, mice were euthanized and blood was collected by puncture of the retro-orbital plexus. Plasma lipoprotein profiles and cholesterol levels were determined using a colorimetric method, according to the manufacturers’ instructions (ABX Pentra, Horiba ABX SAS). Hearts were excised and imbedded in Tissue-Tek OCT (4583, Sakura Finetek) and stored at −80 °C. Serial cryosections (8–10 µm) were performed at the aortic root level for histological staining. For “en face” aorta analysis, the whole aorta was removed by cutting off minor branching arteries and fixed in 4% paraformaldehyde (PFA)/phosphate-buffered saline (PBS). After removing the adventitial and adipose tissue, aortas were cut open longitudinally, pinned out and stained with Oil red O as previously described^18^. Regions analyzed included the aortic arch, thoracic aorta, and abdominal aorta (spanning from the aortic valve to the bifurcation of the iliac arteries).

### Histological and immunofluorescence analyses of atherosclerotic lesions

Lipid content was measured using Oil red O staining of aortic root cryosections. Total collagen content was quantified using Masson’s Trichrome staining. Sirius red staining was used for detection of type I (COL1) and type III (COL3) collagen. PFA-fixed aortic root sections were incubated for 30 min in a freshly prepared 0.1% solution of Sirius Red F3BA (Polysciences Inc., Warrington, Pennsylvania, USA) in saturated aqueous picric acid. Sirius red staining was analyzed using polarization microscopy as described^19^. Immunofluorescence microscopy of frozen-tissue sections was performed as follows: cryosections were fixed in cold methanol (5 min) and acetone (10 min), then washed with PBS, blocked with 1% bovine serum albumin (BSA)/PBS containing 0.3% Triton X-100 for 1 h, and incubated overnight at 4 °C with primary antibodies. After washing in PBS, the sections were incubated for 1 h with an Alexa-546 or -488-conjugated secondary antibody. Nuclei were labeled with 4′,6-diamidino-2-phenylindole (DAPI), and sections were mounted with DAKO fluorescent mounting medium (S3023, DAKO). Studies included parallel experiments omitting primary or secondary antibodies to validate specificity of the antibody binding. The slides were visualized using a Zeiss LSM 780 confocal microscope. To visualize the complete aortic root in one field, we used a 10× objective and Tile Scan acquisition mode. All images were analyzed using Image J software (ImageJ, NIH).

### TUNEL assay

TUNEL assays were performed for the specific detection and quantification of apoptotic cells in cryosections. We used the DeadEnd^TM^ Fluorometric TUNEL System (Promega), which acts by catalytically incorporating fluorescein-12-dUTP at 3’-OH ends of DNA, thus allowing visualization of labeled DNA (in green) directly by fluorescence microscopy. The labeling was performed according to the manufacturer’s recommendations and nuclei were stained with DAPI (blue). A positive control was included by incubating a sample in TACS-Nuclease Buffer (1 h at room temperature). All samples were visualized using a Zeiss LSM 780 confocal microscope, and images were analyzed using Image J software. A Pearson correlation coefficient (PCC) was calculated to quantify the degree of colocalization between green and blue fluorescence, which allows apoptotic cells to be distinguished from debris. Only cells with a PCC ≥ 0.6 were quantified as true apoptotic cells.

### In situ quantification of mitochondrial ROS

To quantify intraplaque mitochondrial ROS production, aortic root cryosections were incubated with MitoSOX Red (5 µM, Invitrogen) for 15 min at room temperature, in darkness. Subsequently, sections were fixed for 15 min in 4% PFA/PBS, counterstained with DAPI and coverslipped using fluorescent mounting medium. Slides were visualized using a Zeiss LSM 780 confocal microscope (excitation 488 nm and emission 580 nm, ×63 magnification) as described^20^.

### Cell culture

VSMCs were isolated from mouse aorta as previously described^8^. Briefly, isolated aortas were incubated with Hank's Balanced Salt Solution (HBSS) containing 1 mg/ml collagenase (Worthington, type II CLS, 4176), 1 mg/mL trypsin inhibitor aprotinin, 0.74 units/mL elastase (Worthington, 2279) and 1% penicillin/streptomycin for 15 min at 37 °C in 5% CO_2_. After stripping the adventitia, the aorta was opened longitudinally and gently scraped to remove blood clots and endothelial cells. The aorta was replaced in a fresh enzyme solution for 1 h and subsequently the cells were centrifuged, washed, and resuspended in Dulbecco’s modified Eagle’s medium/F-12 (DMEM/F-12, Life Technologies) supplemented with 20% fetal calf serum (FCS). VSMCs were used from passages 2 to 7. For each independent experiment, VSMCs were isolated from at least four mice from the same passage number.

Murine VSMCs (MOVAS, from ATCC® CRL-2797) were seeded into a 24-well plate in DMEM containing 10% FCS and 1% penicillin/streptomycin. After 24 h, DMEM was removed and cells were transduced by self-inactivating lentiviral particles at 20 multiplicity of infection, for the expression of plasmids with a gene coding for green fluorescent protein (GFP), a puromycin resistance gene, and an Atg7-targeted or scramble short hairpin RNA (shRNA) gene (Origene, TL504956V). Cells were then incubated at 37 °C for 18 h before replacing the DMEM containing the lentiviral particles by fresh DMEM. Twenty-four hours later, cells of each well were split into a 12-well plate and then DMEM containing 5 µg/mL puromycin (InvivoGen) was added to the wells to maintain a selection pressure. Cells were passed and diluted until puromycin-resistant colonies could be identified.

### LDL isolation and oxidation

LDL from normal human pooled sera were prepared by ultracentrifugation and dialyzed against PBS containing 100 µM EDTA. The LDL pool was then diluted to 2 g/L with PBS into a final volume of 3 mL. LDLs were mildly oxidized by UV-C for 2 h in the presence of 5 µM CuSO_4_ as previously reported^21^. Oxidized LDL contained 4.2–7.4 nmol of TBARS (thiobarbituric acid-reactive substances)/µg apoB. Relative electrophoretic mobility (REM) and 2,4,6-trinitrobenzenesulfonic acid reactive amino groups were 1.2–1.3 times and 85–92 % of native LDL, respectively.

### Western blot analyses

After the treatments, cells were washed in cold PBS and proteins were extracted in solubilizing buffer (10 mM Tris pH 7.4, 150 mM NaCl, 1% Triton X-100, 1% sodium deoxycholate, 0.1% sodium dodecyl sulfate, 1 mM sodium orthovanadate, 1 mM sodium pyrophosphate, 5 mM sodium fluoride, 1 mM phenylmethylsulfonyl fluoride, 1 μg/mL leupeptin, 1 μg/mL aprotinin) for 30 min on ice. For western blot analyses, 30 μg of protein cell extracts were resolved by sodium dodecyl sulfate-polyacrylamide gel electrophoresis and transferred onto polyvinylidene difluoride membranes (Immobilon, IPVH 00010, Millipore). Subsequently, membranes were probed with the indicated primary antibodies and developed with the secondary antibodies coupled to HRP using an ECL chemoluminescence kit (RPN21016, Amersham) and Chemidoc Touch system (Bio-Rad). Membranes were stripped and reprobed with anti-β-actin antibody to control equal loading of proteins. Quantification of the protein bands was performed using Image J software.

### Mitochondrial network imaging

To quantify structural mitochondrial network fragmentation, cells grown on cover glass slides were stimulated as described, then washed with PBS and incubated with MitoTracker Red (1/20,000) for 20 min at 37 °C. Cells were then washed with PBS and fixed in 4% PFA for 10 min. Nuclei were stained with DAPI and cells were mounted with DAKO fluorescent mounting medium. The slides were visualized using a Zeiss LSM 780 confocal microscope. Images were taken at ×63 magnification and a zoom of 3 to visualize the mitochondrial structures. Acquired images were binarized to quantify on cell plans the total mitochondrial area and the average size of mitochondria using Image J software.

### Structured illumination microscopy (SIM)

For high-resolution microscopy experiments, primary VSMCs were grown on special cover glass slides (Marienfilde 1.5 H) and stimulated as described. MitoTracker Red staining was performed as described above. Nuclei were stained with DAPI and cells were mounted with special mounting medium CFM3 with a high refractive index of 1.52 (BioValley). The slides were then visualized using a ZEISS ELYRA PS.1 microscope. Z-stack images were taken every 0.15 µm and reconstructed using a SIM module of Zeiss Zen software. Acquired images were analyzed using Image J software for quantification of total mitochondrial area and average size of mitochondria. Three-dimensional (3D) reconstruction of images and quantification of total mitochondrial volume was performed using IMARIS software.

### Cell immunofluorescence

Cells grown on cover glass slides were fixed in 4% PFA for 10 min, then washed and permeabilized with 0.1% Triton X-100 for 10 min. After blocking with PBS containing 3% BSA for 30 min, cells were incubated with the indicated primary antibodies (1/100) for 1 h and revealed with Alexa Fluor-conjugated secondary antibodies (1/500) for 1 h. Nuclei were labeled with DAPI and cells were mounted with DAKO fluorescent mounting medium. The slides were visualized using a Zeiss LSM 780 fluorescence confocal microscope. All images were analyzed using Image J software.

### Mitochondrial membrane potential and superoxide anion measurement

After the treatments, cells were incubated with JC-1 probe (5 µg/mL) (Enzo Life Sciences, ENZ-52304) for 10 min at 37 °C and then washed twice with PBS. The JC-1 probe is used to determine the level of mitochondrial polarization as it accumulates in healthy mitochondria, selectively generating an orange aggregate emission profile (590 nm), whereas upon mitochondrial injury and loss of membrane potential JC-1 monomers are generated resulting in a shift to green emission (529 nm). The red and green fluorescence levels were determined using a spectrofluorimeter (Tecan) and the ratio of red to green was calculated. Mitochondrial superoxide formation was determined by incubating cells after treatment in the dark with 5 μM MitoSOX Red (M36008, Invitrogen) dye for 30 min and detected at excitation = 510 nm/emission = 580 nm) according to the manufacturer’s recommendations and as described^16^.

### Mitophagy measurements using flow cytometry

Cells plated into six-well plates were stained in complete medium with 10 nM MitoTracker Deep Red (MitoTR) (M22426, Invitrogen) for 15 min at 37 °C, washed and trypsinized for 5 min at 37 °C, then resuspended in PBS. Using an LSRFortessa flow cytometer (Becton Dickinson), 20,000 cells were acquired (FACSDiva Software) and the data were analyzed using the single-cell analysis software FlowJo. The mean fluorescence (FL4 channel) in the viable cell population was plotted and normalized against that of untreated cells as described^22^. Mitophagy flux compares the MitoTR levels with and without lysosomal inhibitors and is calculated as the ratio of MitoTR fluorescence in the presence of lysosomal inhibitors to that in the absence of inhibitors, normalized to the corresponding value in control cells.

### Cell death analysis by SYTO-13/propidium iodide staining

After overnight stimulation, cells were incubated with the permeant DNA intercalating green fluorescent probe SYTO-13 (0.6 µM) and the non-permeant DNA intercalating red fluorescent probe propidium iodide (15 µM) for 20 min at 37 °C as described^23^. Apoptotic and necrotic cells were counted using an inverted fluorescent microscope (Fluovert, Leitz) equipped with a CANON EDS 700D camera. Normal nuclei exhibit green colored diffuse chromatin. Nuclei of primary necrotic cells exhibit red colored chromatin. Apoptotic nuclei exhibit condensed yellow/green colored chromatin associated with nuclear fragmentation, whereas post-apoptotic necrotic cells exhibit the same morphological features, but were red colored. Dead cells were quantified and reported to the total cell number.

### Mitochondrial bioenergetics analysis

Mitochondrial respiration was determined using a Seahorse XF24e extracellular flux analyzer (Seahorse Bioscience) as per the manufacturer’s instructions and as described^24^. Briefly, VSMCs were isolated from mouse aorta as described and were plated into XF24 plates (100777–004, Seahorse Bioscience) at 40.000 cells per well in DMEM/F-12 medium and were allowed to adhere for 24 h. The basal oxygen consumption rate (OCR) was determined, and then oligomycin (1 μM), FCCP (1 μM), and AA/rotenone (1 μM) were sequentially added to measure basal respiration, maximal respiration, ATP production, and spare respiratory capacity. For normalization, cells were lysed in the XF24 plates using protein lysis buffer (50 µL/well) and protein concentration was determined using the Bradford method.

### Statistical analyses

Results are expressed as the mean ± SEM when unpaired Student’s *t*‐test or two-way analysis of variance (ANOVA) followed by Bonferroni post-hoc test were performed. Normality of distribution was assessed using the d’ Agostino & Pearson and Shapiro–Wilk normality tests. When data failed the normal distribution assumption, then non-parametric tests such as Mann–Whitney or Kruskal–Wallis followed by Dunn’s post-hoc test were performed to compare two and multiple groups, respectively, results were expressed as the median with interquartile range. All statistical analyses were performed using GraphPad Prism 6 Software.

## Results

### Autophagy deficiency in VSMCs exacerbates the progression of atherosclerotic lesions

**Fig. 1:**
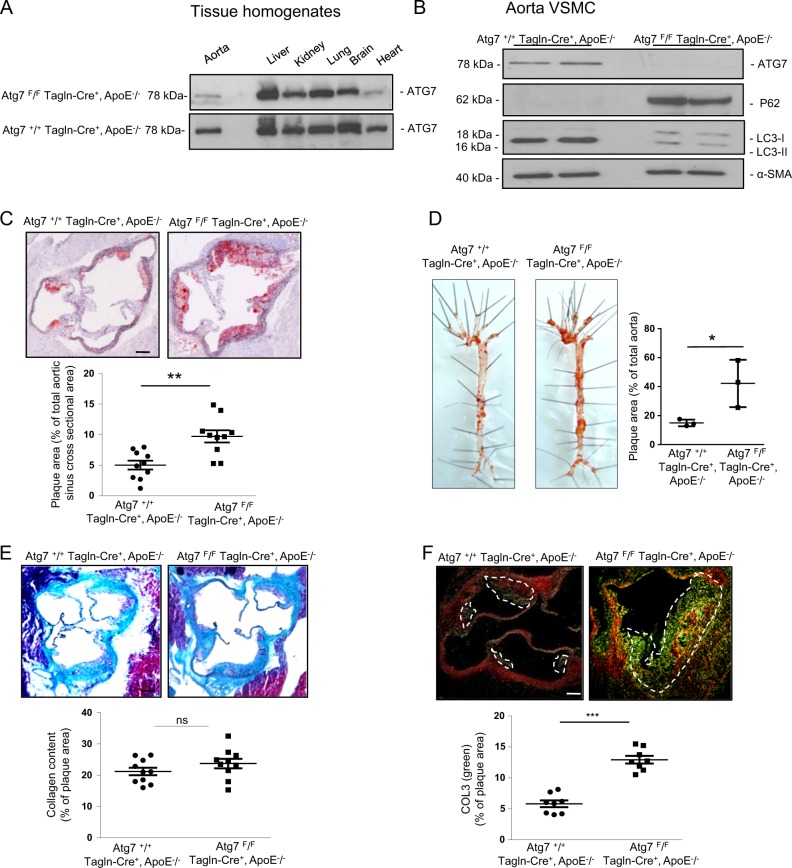
Characterization of Atg7 deletion in *Atg7*^*F/F*^*Tagln-Cre*^*+*^, ApoE^-/-^ tissues and vascular smooth muscle cells (VSMCs), plaque size and type 3 collagen content are increased in ApoE^−/−^ mice deleted for Atg7 in VSMCs. **a** Western blot analyses of ATG7 in different tissue homogenates from *Atg7*^*+/+*^ *Tagln-Cre*^*+*^, ApoE^−/−^ and *Atg7*^*F/F*^ *Tagln-Cre*^*+*^, ApoE^−/−^ mice. **b** Western blot analyses of ATG7, P62, and LC3-I/II protein expression in VSMCs isolated from the aorta of *Atg7* ^*+/+*^ *Tagln-Cre*^*+*^, ApoE^−/−^ and *Atg7*^*F/F*^ *Tagln-Cre*^*+*^, ApoE^−/−^ mice. α-SMA was used as a loading control and is a classical marker for VSMCs. Bands are shown in duplicate for two different primary VSMC cultures. **c** Representative images of consecutive aortic sinus sections stained with oil red after 10 weeks of high-fat diet (HFD) in *Atg7*^*+/+*^ *Tagln-Cre*^*+*^, ApoE^−/−^ and *Atg7*^*F/F*^ *Tagln-Cre*^*+*^, ApoE^−/−^ mice. The graph represents the % of plaque area and the data are the mean ± SEM. **P < 0.01; Student’s *t*-test, *n* = 10 mice/group. Scale bar, 100 µm. **d** Representative images of en face staining of the whole aorta from *Atg7*^*+/+*^ *Tagln-Cre*^*+*^, ApoE^−/−^ and *Atg7*^*F/F*^ *Tagln-Cre*^*+*^, ApoE^−/−^ mice after 18 weeks of HFD. The graph represents the % of plaque area and the data are the mean ± SEM. *P < 0.05; Student’s *t*-test, *n* = 3 mice/group. **e** Representative images of consecutive aortic sinus sections stained with Masson’s trichrome to assess the total collagen content in *Atg7*^*+/+*^ *Tagln-Cre*^*+*^, ApoE^−/−^ and *Atg7*^*F/F*^ *Tagln-Cre*^*+*^, ApoE^−/−^ mice after 10 weeks of HFD. The graph represents the % of total collagen content and the data are the mean ± SEM. ns, nonsignificant; Student’s *t-*test, *n* = 10 mice/group. Scale bar, 100 µm. **f** Representative images of consecutive aortic sinus sections stained with Picrosirius red and analyzed using polarized light microscopy to distinguish between type 1 (red) and 3 (green) collagen after 10 weeks of HFD. The graph represents the % of type 3 collagen content and the data are the mean ± SEM. ***P < 0.001; Student’s *t*-test, *n* = 8 mice/group. Scale bar, 100 µm

**Fig. 2:**
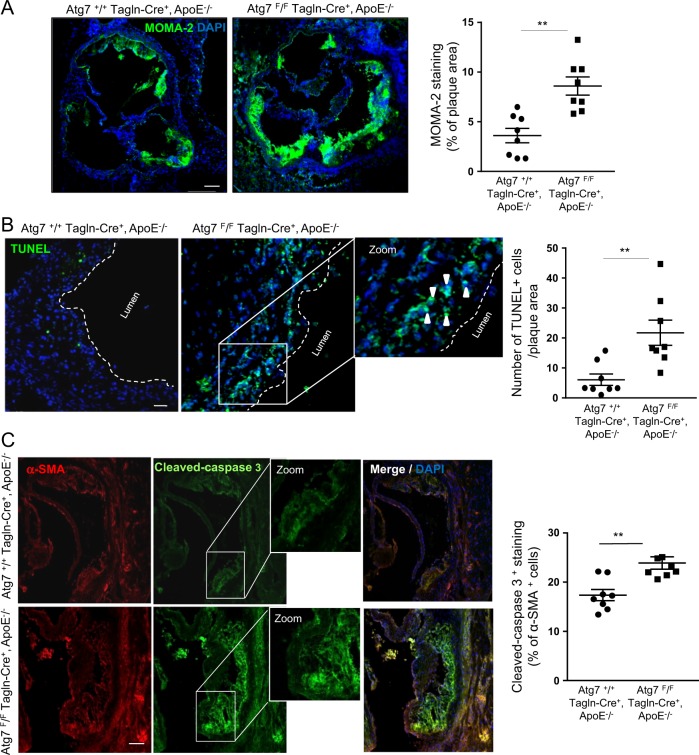
Enhanced inflammation and apoptosis in atherosclerotic lesions of ApoE^−/−^ mice deleted for Atg7 in vascular smooth muscle cells (VSMCs). **a** Representative images of consecutive aortic sinus sections immunostained with MOMA 2 antibody to quantify the content of macrophages/monocytes in the plaque area of *Atg7*^*+/+*^ *Tagln-Cre*^*+*^, ApoE^−/−^ and *Atg7*^*F/F*^ *Tagln-Cre*^*+*^, ApoE^−/−^ mice after 10 weeks of high-fat diet (HFD). DAPI (blue, nucleus). The graph represents the % of MOMA 2 staining and the data are the mean ± SEM, **P < 0.01; Student’s *t*-test, *n* = 8 mice/group. Scale bar, 200 µm. **b** Representative images of apoptotic cell detection by TUNEL staining in aortic sinus sections of *Atg7*^*+/+*^ *Tagln-Cre*^*+*^, ApoE^−/−^ and *Atg7* ^*F/F*^ *Tagln-Cre*^*+*^, ApoE^−/−^ mice after 10 weeks of HFD. TUNEL (green), DAPI (blue, nucleus). The graph represents the number of TUNEL-positive cells measured in the plaque area and the data are the mean ± SEM. **P < 0.01; Student’s *t*-test, *n* = 8 mice/group. Scale bar, 50 µm. **c** Representative images of consecutive aortic sinus sections immunostained with cleaved-caspase 3 (green) and α-SMA (red) antibodies to detect apoptotic VSMCs of *Atg7*^*+/+*^ *Tagln-Cre*^*+*^, ApoE^−/−^ and *Atg7*^*F/F*^ *Tagln-Cre*^*+*^, ApoE^−/−^ mice after 10 weeks of HFD. DAPI (blue, nucleus). The graph represents the % of cleaved-caspase 3-positive VSMCs measured in the plaque area and the data are the mean ± SEM. **P < 0.01; Student’s *t*-test, *n* = 8 mice/group. Scale bar, 50 µm

*Atg7*^*F/F*^*Tagln-Cre*^*+*^ mice harboring a deletion of the essential autophagy gene *Atg7* in VSMCs and their wild-type counterparts (*Atg7*^*+/+*^*Tagln-Cre*^*+*^) were crossbred with ApoE^−/−^ mice to generate *Atg7*^*F/F*^*Tagln-Cre*^*+*^, ApoE^−/−^ and *Atg7*^*+/+*^*Tagln-Cre*^*+*^, ApoE^−/−^ mice, respectively. According to western blot analyses (Fig. 1a) and as expected, we observed decreased expression of ATG7 in aorta and heart tissue homogenates, indeed transgelin or smooth muscle protein 22-alpha is expressed transiently in the heart between E8.0 and E12.5^25^. In isolated *Atg7*^*F/F*^*Tagln-Cre*^*+*^, ApoE^−/−^ VSMCs, the deletion of Atg7 was associated with features of impaired autophagy, including P62/SQSTM1 accumulation and decreased levels of LC3-II as compared with *Atg7*^*+/+*^*Tagln-Cre*^*+*^, ApoE^−/−^ VSMCs (Fig. 1b), thus confirming that the autophagy process is deficient in Atg7-deleted VSMCs. To investigate the consequences of defective VSMC autophagy in the progression of atherosclerosis, *Atg7*^*F/F*^*Tagln-Cre*^*+*^, ApoE^−/−^ and *Atg7*^*+/+*^*Tagln-Cre*^*+*^, ApoE^−/−^ mice were fed a HFD for 10 or 18 weeks. There was no genotype-specific effect on body weight (28.3 ± 0.5 g vs 27.3 ± 0.4 g, *P* = 0.17, *n* = 20 each group) and plasma total cholesterol (25.7 ± 1.9 mM vs 30.7 ± 2 mM, *P* = 0.081, *n* = 20 each group) between *Atg7*^*+/+*^*Tagln-Cre*^*+*^, ApoE^−/−^ and *Atg7*^*F/F*^*Tagln-Cre*^*+*^, ApoE^−/−^ mice after 10 weeks of HFD. Atherosclerotic lesions were significantly increased in *Atg7*^*F/F*^*Tagln-Cre*^*+*^, ApoE^−/−^ mice compared with controls as assayed by both aortic root (10 weeks HFD) and whole aorta *en face* (18 weeks HFD) techniques (Fig. 1c, d). These observations are supported by reports demonstrating the capacity of VSMCs to form foam cells^26^ and the role of autophagy in the regulation of cholesterol efflux from foam cells^27^. Interestingly, while the total collagen showed a similar content in *Atg7*^*F/F*^*Tagln-Cre*^*+*^, ApoE^−/−^ and *Atg7*^*+/+*^*Tagln-Cre*^*+*^, ApoE^−/−^ plaques, the amount of COL3 (less rigid) was significantly increased in *Atg7*^*F/F*^*Tagln-Cre*^*+*^, ApoE^−/−^ plaques (Fig. 1e, f). We also observed an increased macrophage and apoptosis content in *Atg7*^*F/F*^*Tagln-Cre*^*+*^, ApoE^−/−^ plaques as compared with *Atg7*^*+/+*^*Tagln-Cre*^*+*^, ApoE^−/−^ plaques (Fig. 2a, b). In addition, immunohistochemical analyses of aortic sinus sections, using cleaved-caspase 3 and α-SMA (VSMC marker) antibodies, revealed a robust increase in apoptosis activity in *Atg7*^*F/F*^*Tagln-Cre*^*+*^, ApoE^−/−^ plaque VSMCs as compared with wild-type *Atg7*^*+/+*^*Tagln-Cre*^*+*^, ApoE^−/−^ plaques (Fig. 2c). Altogether, these results indicate that autophagy deficiency in VSMCs exacerbates the progression and the unstable phenotype of the atherosclerotic lesions.

### Mitochondrial dysfunction and perturbed mitochondrial quality control in autophagy-deficient VSMCs

**Fig. 3:**
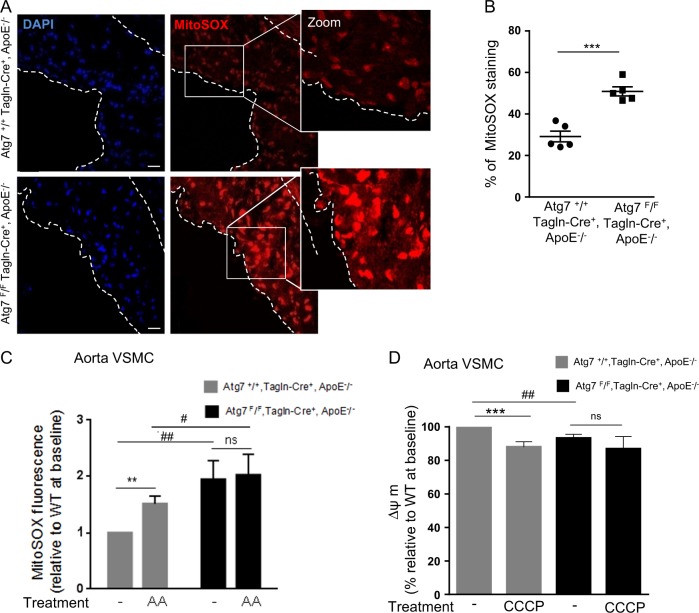
Increased mitochondrial reactive oxygen species (ROS) production and reduced mitochondrial membrane potential in plaques from ApoE^−/−^ mice deleted for Atg7 in vascular smooth muscle cells (VSMCs). **a** Representative images of consecutive aortic sinus sections stained with MitoSOX (red) and DAPI (blue, nucleus) of *Atg7*^*+/+*^ *Tagln-Cre*^*+*^, ApoE^−/−^ and *Atg7*^*F/F*^ *Tagln-Cre*^*+*^, ApoE^−/−^ mice after 10 weeks of high-fat diet (HFD). Scale bar, 20 µm. **b** The graph represents the % of MitoSox staining in the plaque area per section and the data are the mean ± SEM. ***P < 0.001; Student’s *t*-test, *n* = 5 mice/group. **c** Measurement of mitochondrial ROS production in VSMCs isolated from the aorta of *Atg7*^*+/+*^ *Tagln-Cre*^*+*^, ApoE^−/−^ and *Atg7*^*F/F*^ *Tagln-Cre*^*+*^, ApoE^−/−^ mice after 10 weeks of HFD. The graph represents the quantification of MitoSOX Red fluorescence at baseline or after antimycin A (AA, 10 µM) stimulation. Data are the median with interquartile range of four independent experiments from different primary VSMC cultures per group. **P < 0.01; ^##^*P* < 0.01; ^#^*P* < 0.05; ns, nonsignificant; one-way ANOVA, Kruskal–Wallis non-parametric test. **d** Measurement of the mitochondrial membrane potential (ΔΨm) with the JC-1 dye in VSMCs isolated from the aorta of *Atg7*^*+/+*^ *Tagln-Cre*^*+*^, ApoE^−/−^ and *Atg7*^*F/F*^ *Tagln-Cre*^*+*^, ApoE^−/−^ mice after 10 weeks of HFD. The graph represents the quantification of the potential-dependent accumulation of the JC-1 dye in mitochondria at baseline and after CCCP (20 µM) stimulation. Data are the median with interquartile range of four independent experiments from different primary VSMC cultures per group. ***P < 0.001; ^##^*P* < 0.01; ns, nonsignificant; one-way ANOVA, Kruskal–Wallis non-parametric test

Next, we assessed whether the progression of atherosclerosis and plaque instability observed in ApoE^−/−^ mice deleted for Atg7 in VSMCs was associated with dysfunctional and impaired mitochondrial quality control. We first measured ROS production, as excessive mitochondrial ROS contribute to inflammation and cell death. Confocal fluorescence microscopy analyses demonstrated a marked and significant increase in MitoSOX (a mitochondrial targeted fluorescent superoxide sensor) reactivity in *Atg7*^*F/F*^*Tagln-Cre*^*+*^, ApoE^−/−^ lesions, compared with *Atg7*^*+/+*^*Tagln-Cre*^*+*^, ApoE^−/−^ lesions, indicating that autophagy deficiency promotes excessive mitochondrial ROS accumulation (Fig. 3a, b). These results were confirmed in primary cultures of Atg7-deleted aortic VSMCs, which produced enhanced mitochondrial ROS at baseline and after AA treatment, compared with wild-type VSMCs (Fig. 3c).

**Fig. 4:**
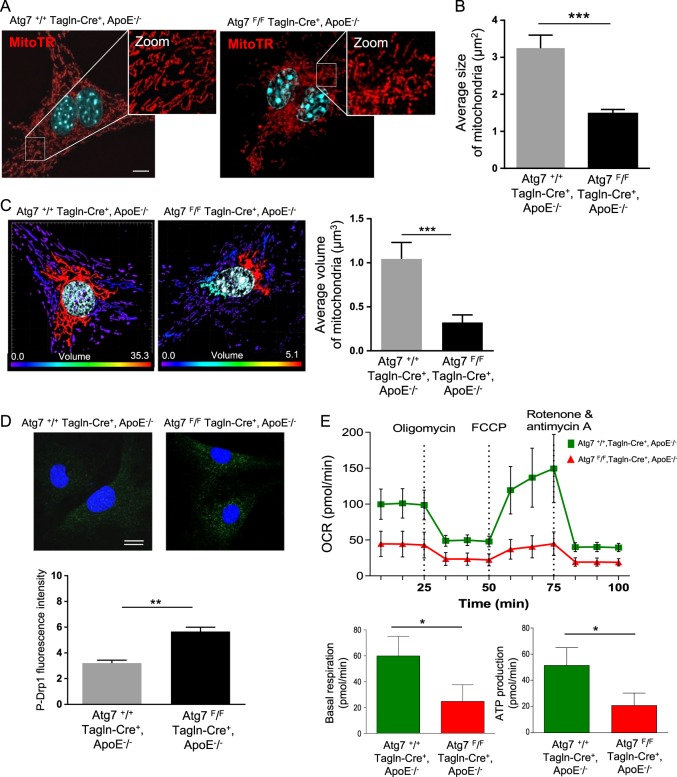
Altered mitochondrial network and bioenergetic functions in ApoE^−/−^ mice deleted for Atg7 in vascular smooth muscle cells (VSMCs). **a** Representative images of aortic VSMCs isolated from *Atg7*^*+/+*^ *Tagln-Cre*^*+*^, ApoE^−/−^ and *Atg7*^*F/F*^ *Tagln-Cre*^*+*^, ApoE^−/−^ mice after 10 weeks of high-fat diet (HFD) and stained with MitoTtracker Deep Red (MitoTR, red), DAPI (blue, nucleus). Scale bar, 5 µm. **b** The graph represents the calculated average size of mitochondria (µm^2^) in aortic VSMCs isolated from *Atg7*^*+/+*^ *Tagln-Cre*^*+*^, ApoE^−/−^ and *Atg7*^*F/F*^ *Tagln-Cre*^*+*^, ApoE^−/−^ mice after 10 weeks of HFD. Data are the mean ± SEM of 10 cells analyzed from three independent experiments from different primary VSMC cultures per group.***P < 0.001; Student’s *t-*test. **c** Representative images of structured illuminated confocal microscopy (SIM) followed by an image processing algorithm deconvolution and 3D reconstruction. The graph represents the calculated average volume of mitochondria (µm^3^) stained with MitoTR, in aortic VSMCs isolated from *Atg7*^*+/+*^ *Tagln-Cre*^*+*^, ApoE^−/−^ and *Atg7*^*F/F*^ *Tagln-Cre*^*+*^, ApoE^−/−^ mice after 10 weeks of HFD. Data are the mean ± SEM of six cells analyzed from two independent experiments from different primary VSMC cultures per group. ***P < 0.001; Student’s *t*-test. **d** Representative images of Ser616 phosphorylated Drp-1 (P-Drp-1, green) and DAPI (blue, nucleus) immunostaining in aortic VSMCs isolated from *Atg7*^*+/+*^ *Tagln-Cre*^*+*^, ApoE^−/−^ and *Atg7*^*F/F*^ *Tagln-Cre*^*+*^, ApoE^−/−^ mice after 10 weeks of HFD. The graph represents the intensity of P-Drp-1 fluorescence. Data are the median with interquartile of six cells analyzed from four independent experiments from different primary VSMC cultures per group. **P < 0.01; Mann–Whitney non-parametric test. **e** Seahorse profile for oxygen consumption rate (OCR) in aortic VSMCs isolated from *Atg7*^*+/+*^ *Tagln-Cre*^*+*^, ApoE^−/−^ and *Atg7*^*F/F*^ *Tagln-Cre*^*+*^, ApoE^−/−^ mice after 10 weeks of HFD with treatment with oligomycin, FCCP, and antimycin A/rotenone. Data are the mean ± SEM of five replicates from three independent experiments from different primary VSMC cultures per group. The graphs represent the basal respiration (last rate measurement before first injection - non mitochondrial respiration rate) and ATP production (last rate measurement before first injection – minimum rate measurement after oligomycin injection) in aortic VSMCs isolated from *Atg7*^*+/+*^ *Tagln-Cre*^*+*^, ApoE^−/−^ and *Atg7*^*F/F*^ *Tagln-Cre*^*+*^, ApoE^−/−^ mice after 10 weeks of HFD, the data are the mean ± SEM of five replicates from three independent experiments from different primary VSMC cultures per group. *P < 0.05; Student’s *t*-test

The mitochondrial membrane potential (ΔΨm) is the main driving force for mitochondrial ATP synthesis and mitochondrial depolarization is an indicator of impaired mitochondrial function. ΔΨm measured in primary VSMCs from *Atg7*^*F/F*^*Tagln-Cre*^*+*^, ApoE^−/−^ mice was significantly decreased, compared with primary VSMCs from *Atg7*^*+/+*^*Tagln-Cre*^*+*^, ApoE^−/−^ mice at baseline, but not under CCCP condition (Fig. 3d). We previously demonstrated that a selective removal of damaged mitochondria through autophagy is athero-protective^16^, thus we investigated whether mitochondrial quality control could be affected by autophagy deficiency. Analyses of the mitochondrial network showed clustered mitochondria around the nucleus and that it was smaller in length in primary VSMCs from *Atg7*^*F/F*^*Tagln-Cre*^*+*^, ApoE^−/−^ mice compared with VSMCs from *Atg7*^*+/+*^*Tagln-Cre*^*+*^, ApoE^−/−^ mice (Fig. 4a). The mitochondrial network from Atg7-deficient VSMCs displayed a globular mitochondrial structure indicative of mitochondrial fragmentation and a marked decrease in the average size of mitochondria (Fig. 4b), supporting the idea that excessive mitochondrial fission produced depolarized mitochondria. Stacks of SIM images, followed by an image processing algorithm deconvolution, allowed the 3D reconstruction of the mitochondrial network in primary VSMCs from *Atg7*^*F/F*^*Tagln-Cre*^*+*^, ApoE^−/−^ mice and from *Atg7*^*+/+*^*Tagln-Cre*^*+*^, ApoE^−/−^ mice. As shown in Fig. 4c, the average calculated volume of mitochondria was significantly smaller in Atg7-deficient VSMCs compared with wild-type VSMCs. Moreover, in primary Atg7-deficient VSMCs we found a significant increase in the cytoplasmic dynamin-related protein 1 (Drp-1) Ser616 phosphorylation, indicating an enhanced activity of the primary regulator of mitochondrial fission (Fig. 4d).

Mitochondrial bioenergic functions were also assessed using a Seahorse extracellular flux analyzer that measures the mitochondrial OCR as an indicator of respiratory reserve capacity after uncoupling electron transport. OCR was significantly reduced both at baseline and after uncoupling in primary VSMCs from *Atg7*^*F/F*^*Tagln-Cre*^*+*^, ApoE^−/−^ mice vs VSMCs from *Atg7*^*+/+*^*Tagln-Cre*^+^, ApoE^−/−^ mice (Fig. 4e). To further validate these observations, immortalized primary vascular aortic smooth muscle cells (MOVAS cells) were transduced with lentiviral shRNAs, targeting the coding region of Atg7 (shAtg7, further referred to as SMC-shAtg7^−/−^). Lentiviral‐mediated knockdown of Atg7 resulted in an efficient reduction of ATG7 protein levels, as compared with a scrambled shRNA control (shSCR, further referred to as SMC-Atg7^+/+^). According to immunoblotting analyses, the knockdown of ATG7 was associated with the accumulation of P62/SQSTM1 and decreased expression of LC3-II in SMC-shAtg7^−/−^, as compared with SMC-Atg7^+/+^ (Supplementary data Fig. S1A). As expected, the processing of LC3-I into LC3-II upon Earle's Balanced Salt Solution (EBSS) or oxidized LDL stimulation was only observed in SMC-Atg7^+/+^ and confirms that SMC-shAtg7^−/−^ are deficient in the initiation of autophagy (Supplementary data Fig. S1A and B). Interestingly, we also observed a significantly fragmented mitochondrial network and a decrease in the average size of mitochondria in SMC-shAtg7^−/−^ compared with SMC-Atg7^+/+^, as well as a significantly diminished ΔΨm (Supplementary data Fig. S1C, D and E).

### Impaired mitophagy and mitochondrial biogenesis exacerbate apoptosis in Atg7-deficient VSMCs

**Fig. 5:**
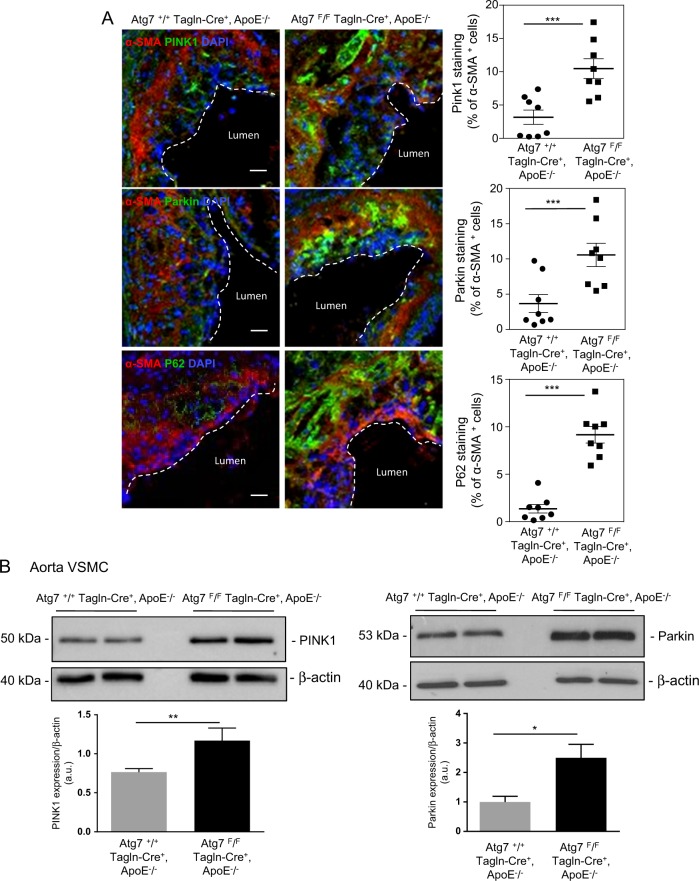
Increased expression of the mitophagy proteins PTEN-induced putative kinase 1 (PINK1) and Parkin functions in VSMCs from atherosclerotic lesions of ApoE^−/−^ mice deleted for Atg7 in vascular smooth muscle cells (VSMCs). **a** Representative images of consecutive aortic sinus sections immunostained with PINK1, Parkin, P62 (red), α-SMA (green) antibodies, and DAPI (blue, nucleus) from *Atg7*^*+/+*^ *Tagln-Cre*^*+*^, ApoE^−/−^ and *Atg7*^*F/F*^ *Tagln-Cre*^*+*^, ApoE^−/−^ mice after 10 weeks of high-fat diet (HFD). The graph represents the % of PINK1, Parkin, or P62 staining in VSMCs within the plaque area and the data are the mean ± SEM from *n* = 8 mice/group. ***P < 0.001; Student’s *t*-test. Scale bar, 20 µm. **b** Western blot analyses of the expression of PINK1 and Parkin proteins in aortic VSMCs isolated from *Atg7*^*+/+*^ *Tagln-Cre*^*+*^, ApoE^−/−^ and *Atg7*^*F/F*^ *Tagln-Cre*^*+*^, ApoE^−/−^ mice after 10 weeks of HFD, β-actin was used as the loading control. Bands are shown in duplicate. The graph represents the densitometric analysis of the expression level of PINK1 and Parkin proteins. The data are the mean ± SEM of three independent experiments from different primary VSMC cultures per group. **P < 0.01; *P < 0.05; Student’s *t*-test

**Fig. 6:**
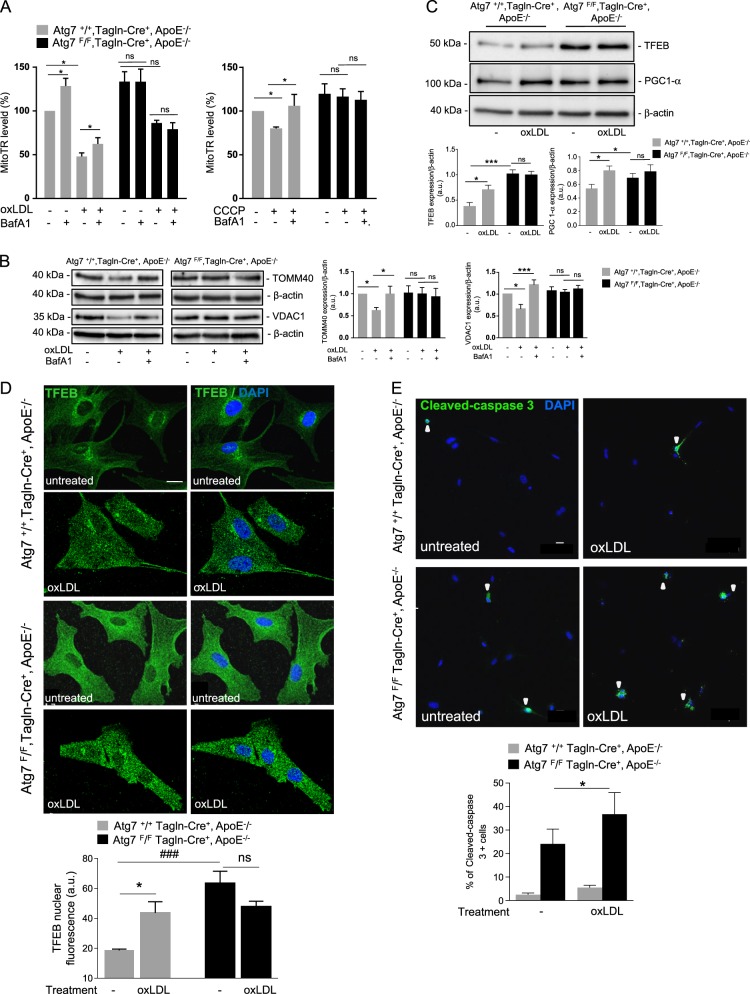
Atg7 deficiency in vascular smooth muscle cells (VSMCs) alters mitochondrial quality control and enhances apoptosis. **a** Flow cytometry analyses of mitophagy flux in aortic VSMCs isolated from *Atg7*^*+/+*^ *Tagln-Cre*^*+*^, ApoE^−/−^ and *Atg7*^*F/F*^ *Tagln-Cre*^*+*^, ApoE^−/−^mice after 10 weeks of high-fat diet (HFD). Cells were incubated either with or without oxidized LDL (200 μg ApoB/mL, left graph) or CCCP (20 µM, right graph) for 8 h and treated either with or without the lysosomal inhibitor Bafilomycin A1 (BafA1) (100 nM). VSMCs were then stained with MitoTR for flow cytometry analysis. The data are expressed as mean ± SEM of five independent experiments (left graph); *P < 0.05; Wilcoxon signed-rank test, ns nonsignificant; and as mean ± SEM of three independent experiments (right graph) *P < 0.05; Mann–Whitney non-parametric test. **b** Western blot analyses of the expression of TOMM 40 and VDAC1 proteins in aortic VSMCs isolated from *Atg7*^*+/+*^ *Tagln-Cre*^*+*^, ApoE^−/−^ and *Atg7*^*F/F*^ *Tagln-Cre*^*+*^, ApoE^−/−^ mice after 10 weeks of HFD. Cells were incubated either with or without oxidized LDL (200 μg ApoB/mL) for 16 h and treated either with or without the lysosomal inhibitor Bafilomycin A1 (BafA1) (100 nM). β-Actin was used as the loading control. The graph represents the densitometric analysis of the expression level of TOMM 40 and VDAC1 proteins. The data are expressed as mean ± SEM of four independent experiments from different primary VSMC cultures. *P < 0.05; ***P < 0.01; one-way ANOVA, Bonferroni’s multiple comparison test. **c** Western blot analyses of the expression of TFEB and PGC-1-α proteins in aortic VSMCs isolated from *Atg7*^*+/+*^ *Tagln-Cre*^*+*^, ApoE^−/−^ and *Atg7*^*F/F*^ *Tagln-Cre*^*+*^, ApoE^−/−^ mice after 10 weeks of HFD. Cells were incubated either with or without oxidized LDL (200 μg ApoB/mL) for 8 h. β-Actin was used as the loading control. The graph represents the densitometric analysis of the expression level of TFEB and PGC-1-α proteins. The data are expressed as mean ± SEM of four independent experiments from different primary VSMC cultures. *P < 0.05; ***P < 0.001; Student’s *t*-test. (**d**) Representative images of TFEB (green) and DAPI (blue, nucleus) immunostaining in aortic VSMCs isolated from *Atg7*^*+/+*^ *Tagln-Cre*^*+*^, ApoE^−/−^ and *Atg7*^*F/F*^ *Tagln-Cre*^*+*^, ApoE^−/−^ mice after 10 weeks of HFD. Cells were incubated either with or without oxidized LDL (200 μg ApoB/mL) for 8 h. The graph represents the intensity of nuclear TFEB fluorescence and the data are the mean ± SEM of three independent experiments from different primary VSMC cultures. *P < 0.05; ^###^*P* < 0.001; one-way ANOVA, Kruskal–Wallis non-parametric test. **e** Representative images of cleaved-caspase 3 (green) and DAPI (blue, nucleus) immunostaining in aortic VSMCs isolated from *Atg7*^*+/+*^ *Tagln-Cre*^*+*^, ApoE^−/−^ and *Atg7*^*F/F*^ *Tagln-Cre*^*+*^, ApoE^−/−^ mice after 10 weeks of HFD. Cells were incubated with or without oxidized LDL (200 μg ApoB/mL) for 16 h. Scale bar, 20 µm. The graph represents the % of cleaved-caspase 3-positive cells and the data are the mean ± SEM of three independent experiments from different primary VSMC cultures. *P < 0.05; two-way ANOVA, Bonferroni’s multiple comparison test. Scale bar, 20 µm

As we previously showed that the molecular mechanism mediating mitophagy in human VSMCs involved the accumulation of PINK1 and the recruitment of the E3 ubiquitin ligase Parkin to mitochondria, we determined the expression levels of PINK1, Parkin, and P62/SQSTM1 proteins in *Atg7*^*F/F*^*Tagln-Cre*^*+*^, ApoE^−/−^ and *Atg7*^*+/+*^*Tagln-Cre*^*+*^, ApoE^−/−^ lesions. The immunoreactivity of PINK1, Parkin, and P62/SQSTM1 was significantly increased in *Atg7*^*+/+*^*Tagln-Cre*^*+*^, ApoE^−/−^ lesions compared with *Atg7*^*F/F*^*Tagln-Cre*^*+*^, ApoE^−/−^ lesions, indicating that the mitophagy process was blocked and this leads to the accumulation of altered mitochondria (Fig. 5a). Consistent with the immunohistochemical results, western blotting analyses revealed that PINK1 and Parkin expression levels were significantly increased in Atg7-deficient VSMCs, compared with wild-type VSMCs (Fig. 5b). We next examined mitophagy flux by flow cytometry in VSMCs from both groups. Cells were stimulated with oxidized LDL or CCCP and treated either with or without Bafilomycin A1, as previously described^16^. Following atherogenic stress or CCCP treatment, mitophagy occurred in VSMCs from *Atg7*^*+/+*^*Tagln-Cre*^*+*^, ApoE^−/−^ mice but not in VSMCs from *Atg7*^*F/F*^*Tagln-Cre*^*+*^, ApoE^−/−^ mice (Fig. 6a). These results were supported by western blot analysis; oxidized LDL treatment decreased the levels of mitochondrial proteins including TOMM 40 and VDAC1 in VSMCs from *Atg7*^*+/+*^*Tagln-Cre*^*+*^, ApoE^−/−^ mice but not in VSMCs from *Atg7*^*F/F*^*Tagln-Cre*^*+*^, ApoE^−/−^ mice (Fig. 6b). These findings raised the question whether the alteration in mitophagy is also coupled to an impaired compensatory mitochondrial biogenesis, causing dysfunctional mitochondrial quality control. Peroxisome proliferator-activated receptor gamma coactivator 1-alpha (PGC-1α) is the master regulator of mitochondrial biogenesis, whereas transcription factor EB (TFEB) regulates lysosomal biogenesis. TFEB reciprocally regulates PGC-1α expression to enhance compensatory mitochondrial biogenesis to replenish the mitochondrial pool removed by mitophagy^28^. Thus, we examined the expression levels of PGC-1α and TFEB in primary VSMCs stimulated with the mitophagy inducer oxidized LDL. As shown in Fig. 6c, d, the increased expression of PGC-1α and TFEB proteins measured in stimulated-wild-type VSMCs, was blunted in Atg7-deficient VSMCs, as well as the increase in the nuclear translocation of TFEB upon oxidized LDL stimulation. Of note, we observed at baseline in Atg7-deficient VSMCs, a higher level of total TFEB expression in addition to its nuclear expression if compared with wild-type VSMCs. This observation suggests that Atg7-deficient VSMCs try other ways to activate the autophagy machinery and/or the production of energy because TFEB is strongly involved in the transcription of autophagy genes. Furthermore, cleaved-caspase 3 immunostaining was significantly enhanced in Atg7-deficient VSMCs treated with oxidized LDL compared with wild-type VSMCs, indicating that a dysfunctional mitochondria quality control aggravates atherogenic stress-induced VSMC apoptosis (Fig. 6e). Similar results were obtained in SMC-shAtg7^-/-^, which display increased susceptibility to apoptotic inducers (Supplementary data Fig S2A and B).

## Discussion

Despite recent advances in medical and interventional therapies, CVD continues to be the leading cause of death worldwide. Atherosclerosis is—by far—the main cause of most CVD. This progressive and complex disease is often associated with the aging process and involves a build-up of deposits in the arterial wall, called an atherosclerotic plaque. The composition, rather than the plaque size or severity of stenosis, plays a critical role in plaque rupture and thrombosis. Consequently, critical mechanisms related to the establishment and the progression of this disease need to be extensively investigated. The mechanisms involved in plaque stability and plaque rupture are complex and depend on the cell survival vs death balance of the cellular components of the lesion, such as VSMC apoptosis, which is a common feature of high-risk/vulnerable atherosclerotic plaques.

Basal autophagy is a necessary process for proper vascular function and wide-spread evidence indicates that autophagy is also stimulated by stress-related stimuli in the vascular wall^7^. Recently, we demonstrated that successful autophagy of dysfunctional mitochondria stimulates VSMC survival^16^, whereas reduced autophagy promotes age-related changes in the vasculature^2^. However, it remained unclear whether reduced autophagic flux affects the mitochondrial quality control of VSMCs in atherosclerotic plaques. In this study, by using an in vivo model of atherosclerosis with a deletion of the autophagy gene *Atg7* in VSMCs, we provide evidence that a worsened apoptotic and inflammatory phenotype of the plaques occurred compared with their wild-type counterparts. Our observations are in line with previous reports showing that defective autophagy accelerates atherogenesis^8^. Interestingly, our study reveals several new important findings regarding mitochondrial status both in vivo and in vitro. Mitochondria are crucial for many cellular functions including ATP generation, redox balance, calcium stores, and cell death. Mitochondrial turnover is an essential aspect of the mitochondrial quality control process, in which dysfunctional mitochondria are selectively eliminated through autophagy (mitophagy) and replaced through expansion of preexisting mitochondria (biogenesis)^29^. Even though recent evidence has demonstrated that knocking out genes involved in the formation of autophagosomes results in the accumulation of damaged mitochondrial and the development of cardiac dysfunction in mice^30–32^, no information is available from atherosclerotic vessels, especially in VSMCs. This study is the first to demonstrate that in a mouse model of atherosclerosis, impaired autophagy in VSMCs exhibits disrupted mitochondrial quality control, which is characterized by the accumulation of fragmented mitochondria with reduced bioenergetic efficiency and more oxidative stress. These results suggest a defect in the recognition of altered mitochondria and uptake by lysosomes. Indeed, we detected both in plaques and mouse VSMCs an accumulation of p62/SQSTM1, as well as PINK1 and Parkin, which are involved in the molecular mechanisms mediating mitophagy^16^. These data are strengthened by the inhibition of mitophagic flux measured in Atg7-deleted VSMCs and supports the concept that disrupted autophagic flux, along with the accumulation of dysfunctional mitochondria, is linked to impaired mitophagy. Moreover, given that successful mitophagy protects against oxidative stress and the release of proteins that participate in cell death pathways, we observed increased apoptosis in plaques of Atg7^F/F^ Tagln/SM22α Cre^+^ mice and in Atg7-deleted VSMCs. Mitochondrial biogenesis and mitophagy are linked in both directions. PGC-1α, regulating mitochondrial biogenesis, induces expression of TFEB, a master regulator of lysosome biogenesis and autophagy^28,33^. Although wild-type VSMCs stimulated with oxidized LDL display increased expression of PGC-1α and TFEB, as well as its nuclear translocation, this process is lacking in Atg7-deleted VSMCs. However, the higher level of TFEB expression at baseline in Atg7-deleted VSMCs suggests that the cells try to counteract the autophagy deficiency and/or the production of energy by other ways. Indeed, TFEB orchestrates the transcription of genes involved in autophagy and lysosomal exocytosis. Therefore, the accumulation of dysfunctional mitochondria in ApoE^−/−^ mice with a VSMC-specific deletion of Atg7 likely results from impaired clearance of damaged organelles by autophagy, as well as the inadequate replenishment of the cellular mitochondrial pool by mitochondrial biogenesis.

Taken together, these results indicate that a loss of autophagic flux is detrimental to the maintenance of a healthy mitochondrial population, which contributes to VSMC apoptosis and, in turn, leads to necrotic core formation and a decreased fibrous cap in atherosclerotic plaques. Our findings also underline the relevance of autophagy dysfunction in vascular disorders and raises the therapeutic interest of improving mitochondrial quality control as a promising strategy to stabilize atherosclerotic plaques. The list of potential pharmacological agents inducing cellular mitophagy have been recently reviewed^34^, however, the conventional pharmacological approaches to initiating mitophagy in vitro reside in the use of agents that induce the dissipation of the mitochondrial ΔΨm or impair mitochondrial respiration, which limits their possible use in vivo and in clinic. Since we previously demonstrated that the overexpression of PINK1 and Parkin in human VSMCs was protective by limiting cell death and potentiating mitophagy^16^, we can consider that the genetically or pharmacologically rescue of mitophagy through enhancing the PINK1/Parkin pathway could be an efficient alternative. For instance, the 18-kDa translocator protein (TSPO), has been shown to regulate mitophagy downstream of the PINK1/Parkin pathway without interfering directly with the pathway but through a ROS-sensitive mechanism^35^. Notably, the expression level of TSPO is elevated in pathological situations linked to mitophagy defects thus highlighting its pharmacological interest as a potential target to therapeutically activate mitophagy. Because the TSPO levels in vascular disease conditions such as atherosclerosis remain unknown, it will be therefore valuable to investigate its expression. More generally, there is a need to devise pharmacological tools specifically conceived to modulate mitophagy without perturbing the organelle.

## Supplementary information

Supp data
