# Supplementary material for: Altered mitochondrial quality control in Atg7-deficient VSMCs promotes enhanced apoptosis and is linked to unstable atherosclerotic plaque phenotype
Source: Cell Death Dis. 2019 Feb 11;10(2):119. doi: 10.1038/s41419-019-1400-0 (PMC6370858; doi:10.1038/s41419-019-1400-0)
Supplement: Supplementary file 1 — Supp data [file 41419_2019_1400_MOESM1_ESM.pdf]

Supplementary data Figure S1

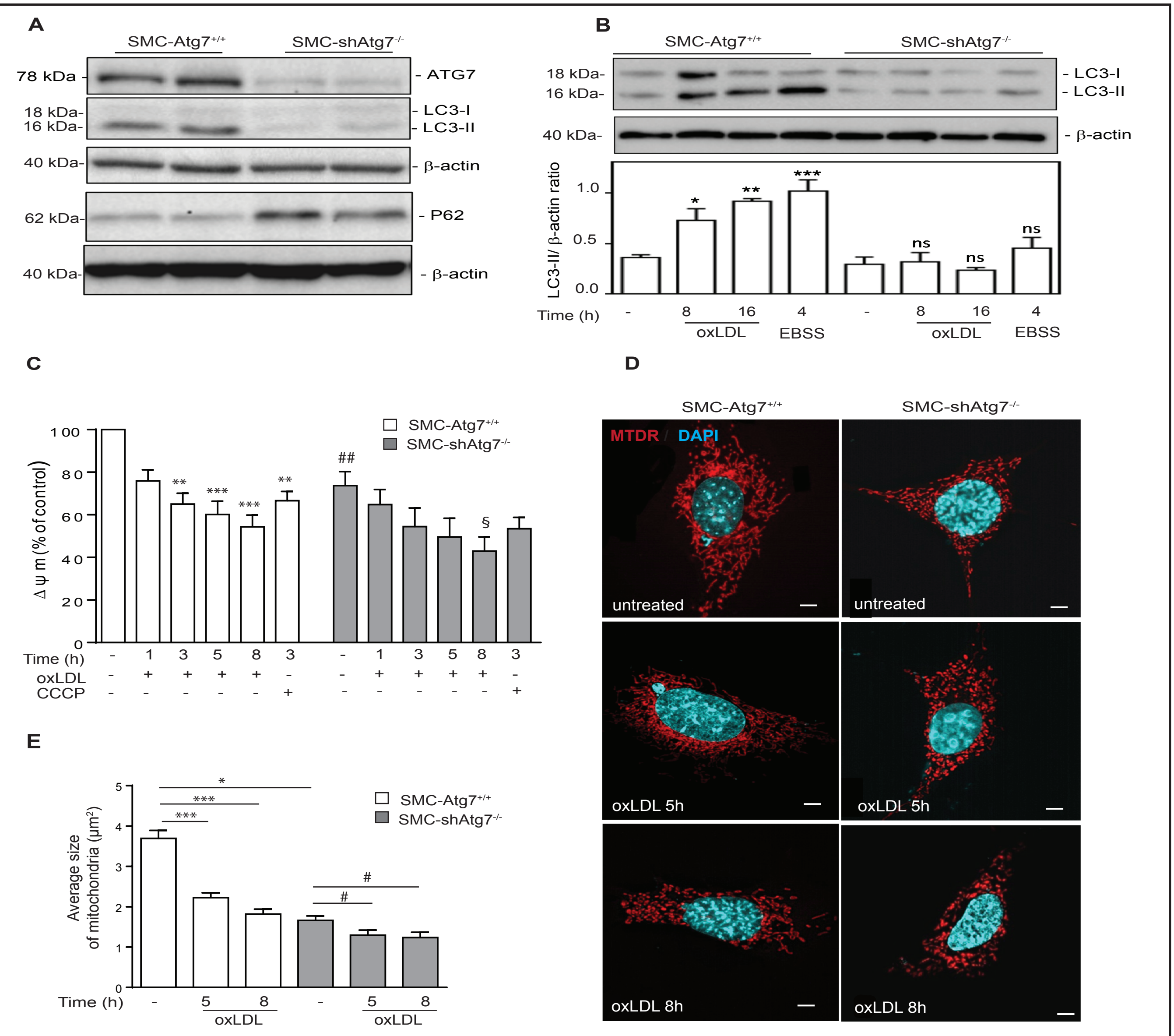

Figure S1

(**A**) Western blot analysis of ATG7, P62 and LC3I/II protein expression levels in SMC-$Atg7^{+/+}$ and SMC-$shAtg7^{-/-}$ lysates. β-actin was used as a loading control. Bands are shown in duplicate for two different cell cultures.

(**B**) Western blot analyses of LC3-I to LC3-II conversion in SMC-$Atg7^{+/+}$ and SMC-$shAtg7^{-/-}$ lysates. Cells were stimulated either with or without oxidized LDL (oxLDL, 200 µg ApoB/ml) at the indicated time or with EBSS for 4h. Blots are representative of 3 independent experiments. The graph represents values (means ± SEM) of LC3-II band intensity after normalization for β-actin by densitometry. * $P < 0.05$, ** $P < 0.01$,*** $P < 0.001$; Student's t test, ns indicates no significance.

(**C**) Measurement of the mitochondrial membrane potential (ΔΨm) with the JC-1 dye in SMC-$Atg7^{+/+}$ and SMC-$shAtg7^{-/-}$ cells after treatment either with or without oxidized LDL (oxLDL, 200 µg ApoB/ml) or CCCP (20 µM) at the indicated times. The graph represents the quantification of the potential-dependent accumulation of the JC-1 dye in mitochondria. The data are expressed as a mean ± SEM of 9 independent experiments , ** $P < 0.01$,*** $P < 0.001$, ## $P < 0.01$, § $P < 0.05$, one-way ANOVA, Tukey's multiple comparison test.

(**D**) Representative images of structure illuminated confocal microscopy (SIM) from SMC-$Atg7^{+/+}$ and SMC-$shAtg7^{-/-}$ cells after treatment either with or without oxidized LDL (oxLDL, 200 µg ApoB/ml) at the indicated times and stained with (MitoTR, red), DAPI (blue, nucleus). Scale bar, 5 µm.

(**E**) The graph represents the calculated average size of mitochondria (µm²) of SMC-$Atg7^{+/+}$ and SMC-$shAtg7^{-/-}$ cells after treatment either with or without oxidized LDL (oxLDL, 200 µg ApoB/ml) at the indicated times. The data are expressed as a mean ± SEM of 3 independent experiments; *** $P < 0.001$, # $P < 0.05$, two-way ANOVA, Bonferroni's multiple comparison test.

Supplementary data Figure S2

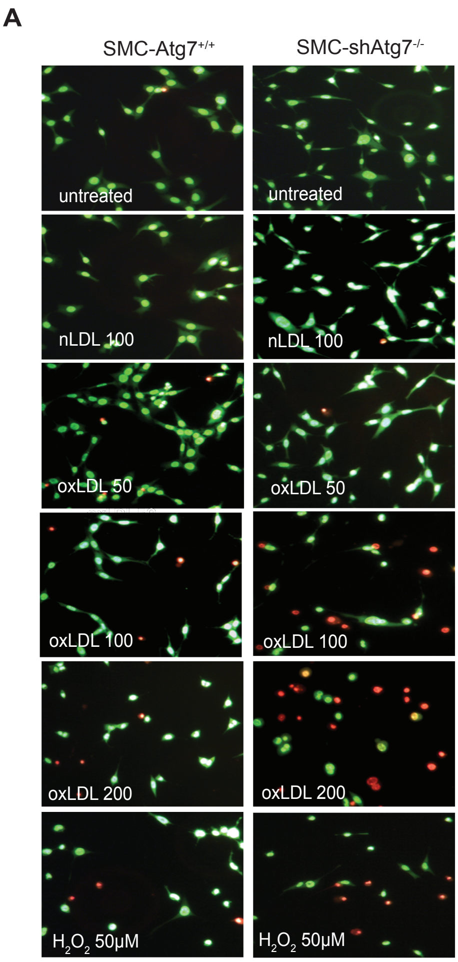

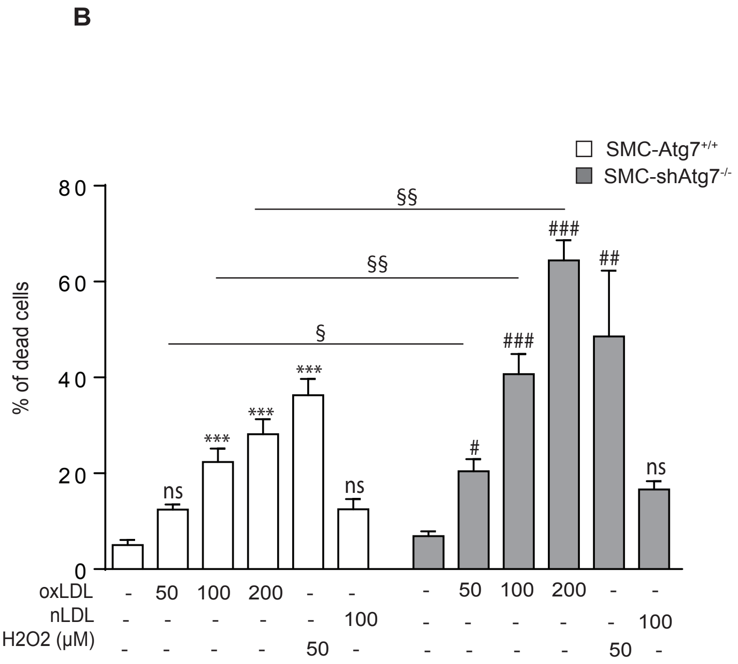

Figure S2
(**A**) Representative images of SMC-Atg7+/+ and SMC-shAtg7-/- cell viability after various treatments as indicated. Cells were stained with SYTO-13/PI and analyzed using an inverted microscope as described in 'Materials and Methods.'
(**B**) The graph represents the results expressed as a percentage of dead cells after the different treatments: oxidized LDL (oxLDL, 50, 100 or 200 µg ApoB/ml), native LDL (nLDL, 100 µg ApoB/ml) or H_2O_2 50 µM during 16h (>200 cells were counted for each variable per experiment). The data are expressed as the mean ± SEM of 6 independent experiments. *** $P < 0.001$, ### $P < 0.001$, ## $P < 0.01$, § $P < 0.05$, §§ $P < 0.01$, ns indicates no significance. One-way ANOVA, Tukey's multiple comparison test.
